# Miscellany

**Published:** 1908-03

**Authors:** 


### MISCELLANY.

ARMY MEDICAL CORPS EXAMINATIONS.—Preliminary examinations for appointment of Assistant Surgeons in the Army will be held on May 4 and August 3, 1908, at points to be hereafter designated. Full information concerning the examination can be procured upon application to the Surgeon General, U. S. Army, Washington, D. C. The essential requirements to securing an invitation are that the applicant shall be a citizen of the United States, shall be between twenty-two and thirty years of age, a graduate of a medical school legally authorised to confer the degree of doctor of medicine, shall be of good moral character and habits, and shall have had at least one year's hospital training or its equivalent in practice.

The examinations will be held concurrently throughout the country at points where boards can be convened. Due consideration will be given to the localities from which applications are received, in order to lessen the traveling expenses of applicants as much as possible. Applicants holding diplomas from reputable literary or scientific colleges, normal schools or high schools, or graduates of medical schols which require an entrance examination satisfactory to the faculty of the Army Medical School, will not be examined in subjects of general preliminary education.

In order to perfect all necessary arrangements for the examinations of May 4th, applications must be complete and in possession of the Surgeon General on or before April 1st. Early attention is therefore enjoined upon all intending applicants. There are at present twenty-three vacancies in the Medical Corps of the Army.

